# Supplementary material for: Connectome analysis of male world‐class gymnasts using probabilistic multishell, multitissue constrained spherical deconvolution tracking
Source: J Neurosci Res. 2021 Jul 10;99(10):2558–72. doi: 10.1002/jnr.24912 (PMC9541483; doi:10.1002/jnr.24912)
Supplement: Supplementary file 3 — TABLE S1 Interpretations of graph metrics TABLE S2 Cohen’s d of each global metric across the full range of sparsity thresholds for comparison between the world‐class gymnasts and controls TABLE S3 Networks identified as significantly different between world‐class gymnasts and controls using network‐based statistical analysis TABLE S4 Network identified as significantly different between world‐class gymnasts and controls using network‐based statistical analysis [file JNR-99-2558-s003.docx]

**Table S1. Interpretations of graph metrics**

| **Metric** | **Interpretation** |
| --- | --- |
| **Local network metrics** | |
| **Nodal strength** | The simplest measure of ***centrality*** (functional importance) of a given node. The nodal strength is the sum of weights of links connected to the node (Watts and Strogatz, 1998). |
| **Nodal degree** | The greater the degree of a node, the more important it might be in the brain network. The nodal degree is the number of edges connected to the node. |
| **Betweenness centrality** | Betweenness centrality also measures the ***centrality*** (functional importance) of a node, but it reflects the importance of the node in the information transfer within the brain network (Freeman, 1979). |
| **Local clustering** | Local clustering measures the ***functional segregation*** of a node, which reflects the degree of clustered connectivity around a given node (Rubinov and Sporns, 2010; Watts and Strogatz, 1998). |
| **Local efficiency** | Local efficiency also measures ***functional segregation*** of a node, which measures the ability of the node to transfer information among its neighbors (Rubinov and Sporns, 2010). |
| **Global network metrics** | |
| **Mean strength** | Mean strength is the average of the nodal strength of all nodes within a brain network. |
| **Global clustering** | Global clustering is the average of the local clustering of all nodes within a brain network, which measures the ***functional segregation*** of a network. This represents the penetration of clustered connectivity around individual nodes (Rubinov and Sporns, 2010; Watts and Strogatz, 1998). |
| **Global efficiency** | **Global efficiency** measures the ***functional integration*** of a network, which reflects its ability to exchange information efficiently (Rubinov and Sporns, 2010). |
| **Characteristic path length** | Characteristic path length is the most common measure of ***functional integration,*** which reflects the capability to transfer information in parallel across the whole brain network (Rubinov and Sporns, 2010). |
| **Small-world property** | Small-world networks are defined as networks that have a small average shortest path length but also a clustering coefficient significantly higher than expected by random networks (Watts and Strogatz, 1998). A small-world property ($\sigma$) > 1 indicates that the network has small-worldness (Humphries and Gurney, 2008). |

**References:**

Freeman, L.C. (1979). Centrality in social networks conceptual clarification. Soc Networks 1:215-239.

Humphries, M.D., Gurney K. (2008). Network "small-world-ness": a quantitative method for determining canonical network equivalence. *PloS one* 3(4):e0002051. doi: 10.1371/journal.pone.0002051

Rubinov M., Sporns O. (2010). Complex network measures of brain connectivity: uses and interpretations. *NeuroImage* 52:1059-1069. doi: 10.1016/j.neuroimage.2009.10.003

Watts D.J., Strogatz S.H. (1998). Collective dynamics of "small-world" networks. *Nature* 393:440-442. doi: 10.1038/30918

**Table S2. Cohen’s *d* of each global metric across the full range of sparsity thresholds for comparison between the world-class gymnasts and controls**

|  | **Partial eta-squared** | | | | | | |
| --- | --- | --- | --- | --- | --- | --- | --- |
| **Threshold** | 100% | 5% | 10% | 15% | 20% | 25% | 30% |
| Mean strength | 0.55 | 0.56 | 0.54 | 0.54 | 0.54 | 0.54 | 0.54 |
| Global clustering | 0.0020 | 0.0050 | 0.039 | 0.045 | 0.045 | 0.049 | 0.038 |
| Global efficiency | 0.054 | 0.061 | 0.055 | 0.054 | 0.054 | 0.054 | 0.054 |
| Characteristic path length | 0.47 | 0.46 | 0.44 | 0.47 | 0.47 | 0.47 | 0.47 |
| Small-world property | 0.16 | 0.18 | 0.22 | 0.21 | 0.20 | 0.20 | 0.20 |

**Table S3. Networks identified as significantly different between world-class gymnasts and controls using network-based statistical analysis**

| *P* = 0.05  T = 1.75 | **Network 1**  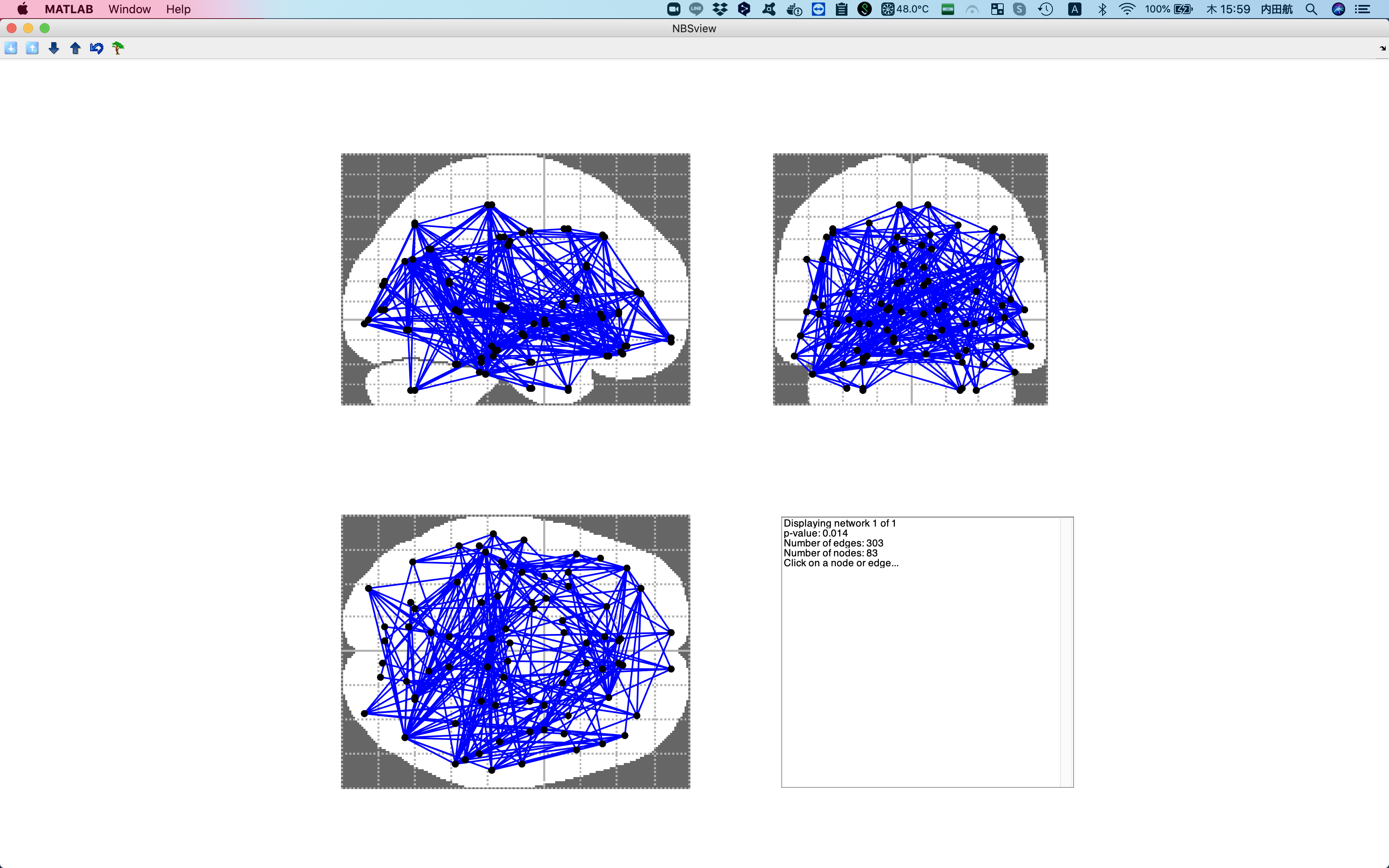 |
| --- | --- |
| *P* = 0.02  T = 2.24 | **Network 1**  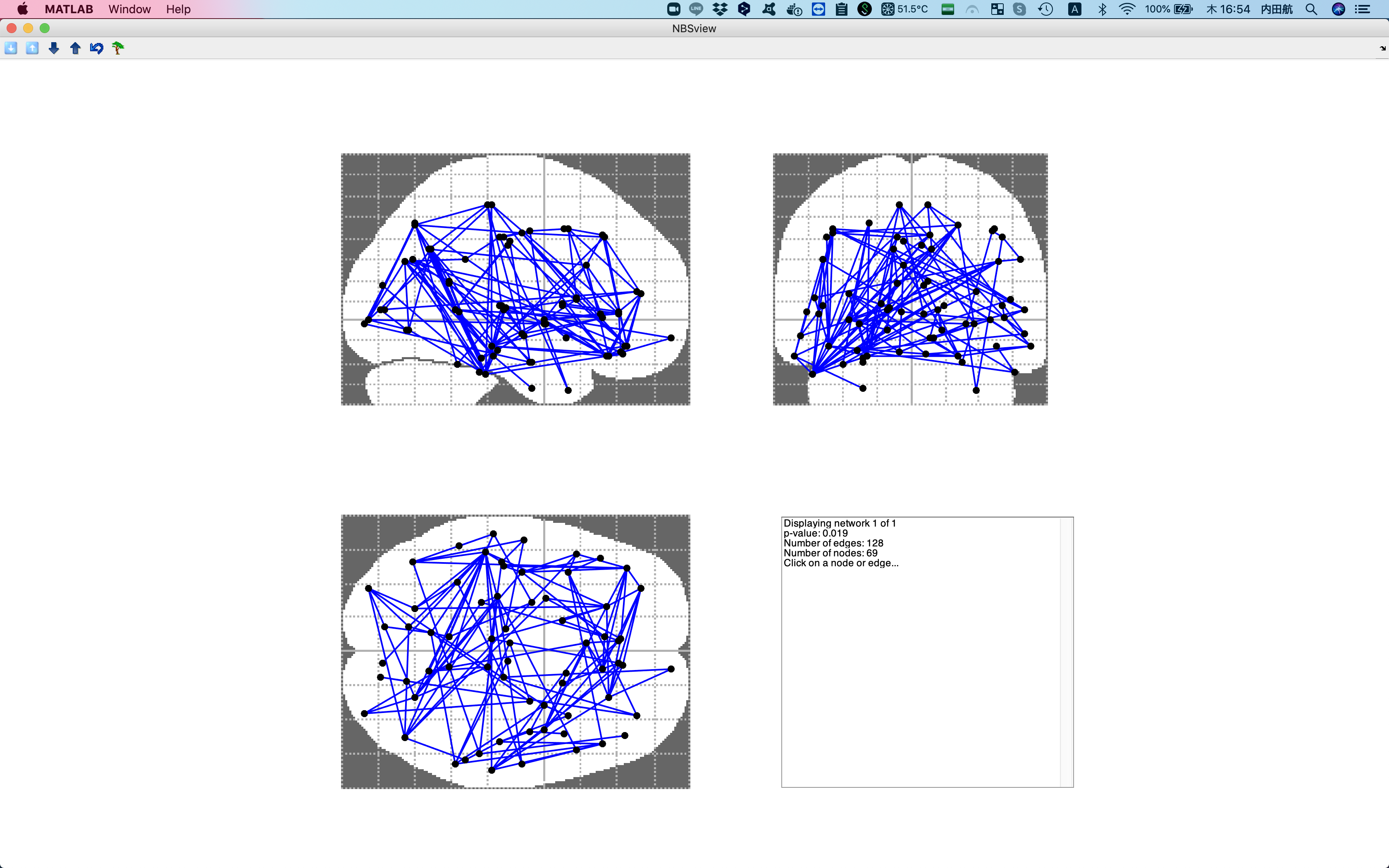 |
| *P* = 0.01  T = 2.58 | **Network 1**  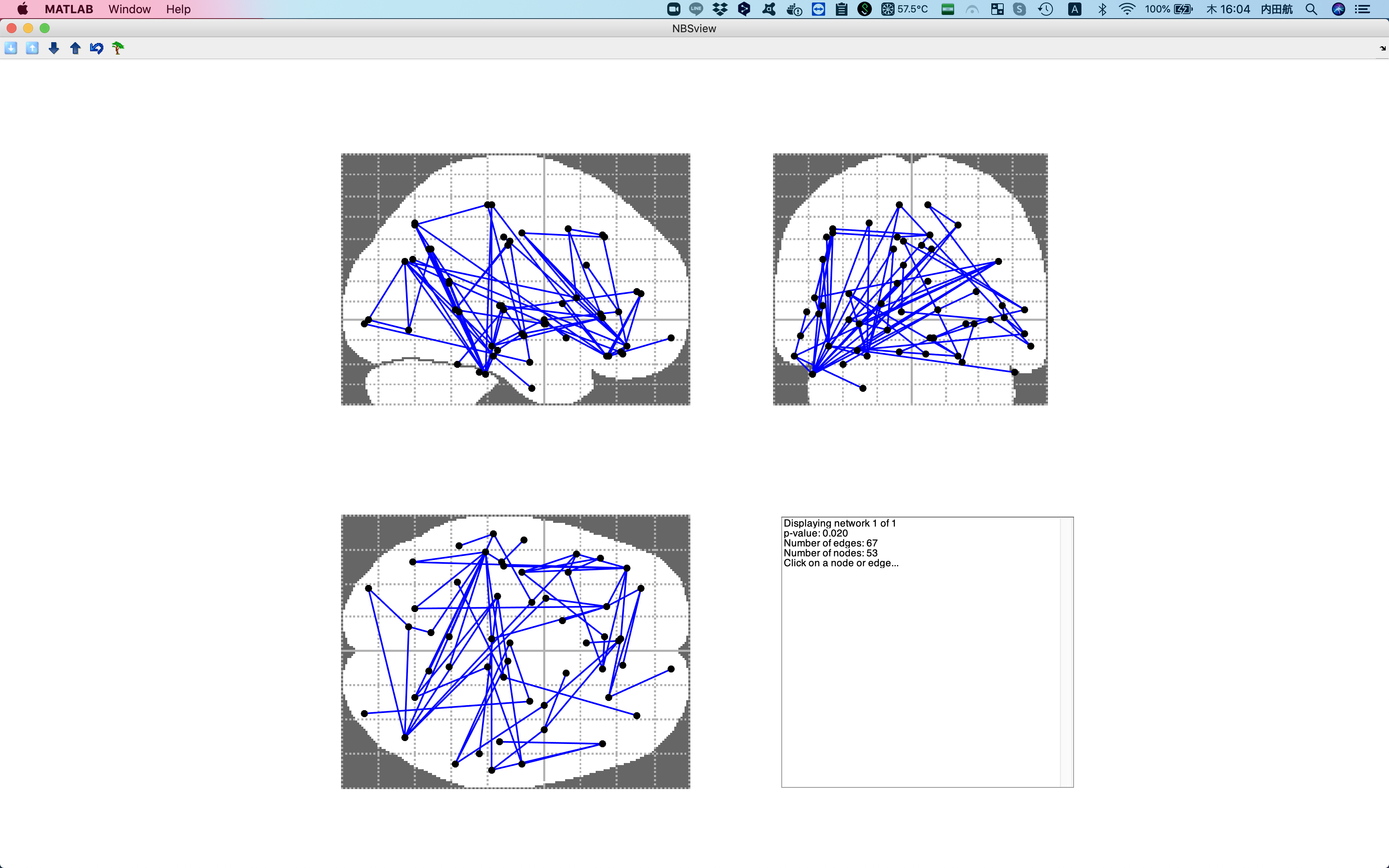 |
| *P* = 0.005  T = 2.92 | **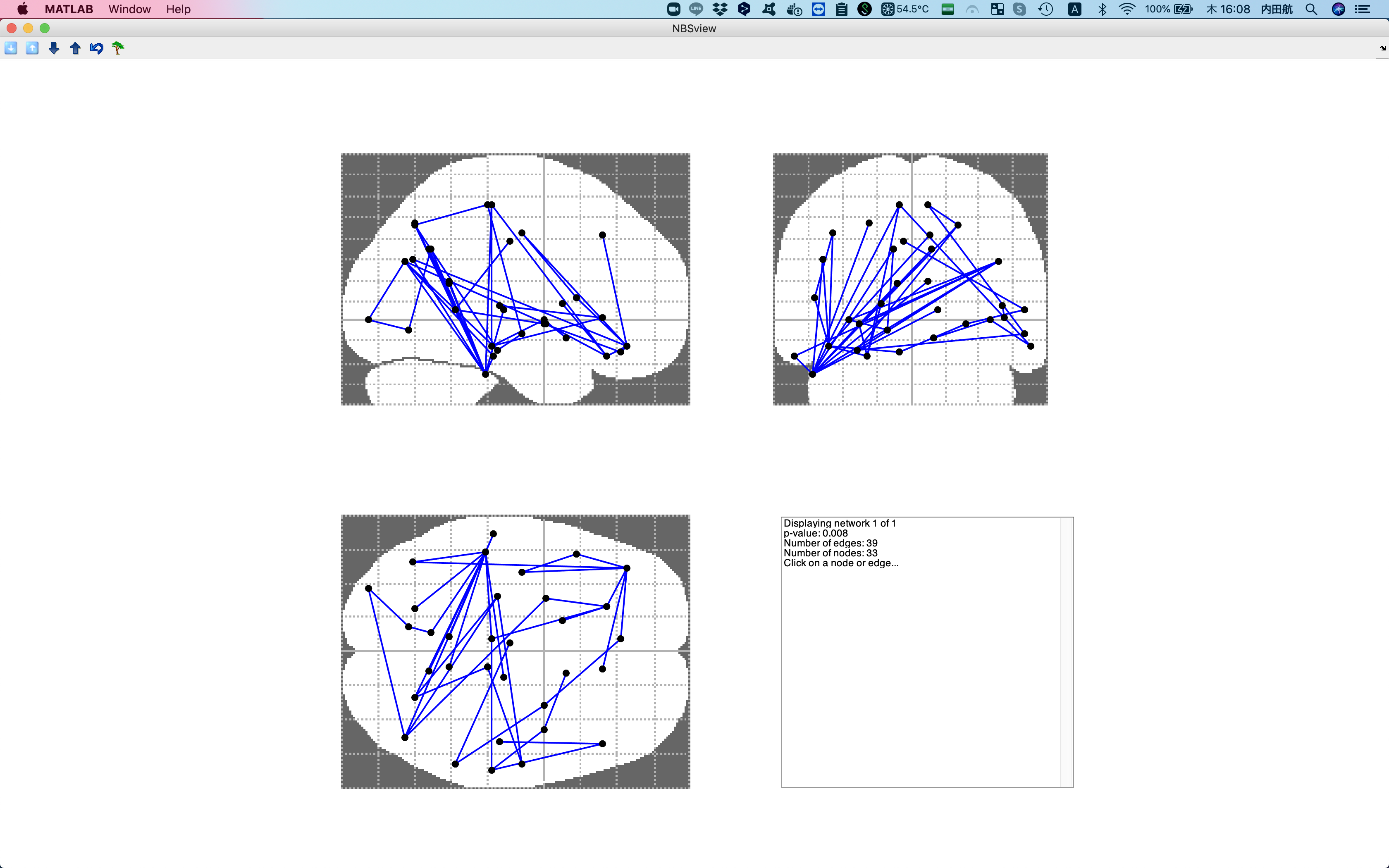** |

We explored the results of the network-based statistic using different thresholds to individuate the suprathreshold edges (0.05 ≤ *P* ≤ 0.001).

**Table S4. Network identified as significantly different between world-class gymnasts and controls using network-based statistical analysis**

| **Identified connection (*P* = 0.020)** | ***t* -value** |
| --- | --- |
| Left inferior parietal to right middle temporal | 5.49 |
| Right inferior temporal to right paracentral | 5.46 |
| Left superior parietal to right hippocampus | 4.03 |
| Right lingual to right precuneus | 3.97 |
| Left inferior parietal to right hippocampus | 3.92 |
| Left isthmus cingulate to right inferior temporal | 3.88 |
| Left thalamus proper to right inferior temporal | 3.86 |
| Right lateral occipital to right lingual | 3.71 |
| Left paracentral to left superior temporal | 3.67 |
| Left inferior parietal to right putamen | 3.56 |
| Right pars opercularis to right precentral | 3.50 |
| Left superior frontal to right pars orbitalis | 3.49 |
| Left insula to left accumbens area | 3.47 |
| Left bankssts to right posterior cingulate | 3.47 |
| Right pars orbitalis to right precentral | 3.45 |
| Right lateral orbitofrontal to right paracentral | 3.42 |
| Right inferior temporal to right isthmus cingulate | 3.40 |
| Left middle temporal to right paracentral | 3.38 |
| Right medial orbitofrontal to right pars orbitalis | 3.38 |
| Right lateral orbitofrontal to right pars orbitalis | 3.36 |
| Left pars triangularis to left transverse temporal | 3.35 |
| Left lateral orbitofrontal to right rostral middle frontal | 3.34 |
| Left paracentral to left superior parietal | 3.32 |
| Left inferior parietal to right inferior temporal | 3.27 |
| Right caudal middle frontal to right pars triangularis | 3.27 |
| Left superior parietal to right inferior temporal | 3.25 |
| Left precuneus to right inferior temporal | 3.23 |
| Left superior temporal to right hippocampus | 3.20 |
| Right inferior parietal to right inferior temporal | 3.15 |
| Left putamen to right medial orbitofrontal | 3.14 |
| Right superior temporal to right transverse temporal | 3.14 |
| Left bankssts to left putamen | 3.12 |
| Right inferior parietal to right pars orbitalis | 3.06 |
| Right inferior temporal to right superior parietal | 3.04 |
| Left middle temporal to left insula | 3.03 |
| Right inferior temporal to right precuneus | 3.02 |
| Right inferior temporal to right middle temporal | 2.99 |
| Right pars opercularis to right pars orbitalis | 2.98 |
| Right putamen to right lateral orbitofrontal | 2.96 |
| Left inferior parietal to right lateral occipital | 2.95 |
| Left middle temporal to left pars triangularis | 2.94 |
| Right caudate to right lateral orbitofrontal | 2.93 |
| Left pars triangularis to left superior temporal | 2.92 |
| Right lateral orbitofrontal to right superior parietal | 2.91 |
| left rostral caudal middle frontal to left thalamus proper | 2.84 |
| Left insula to right rostral anterior cingulate | 2.82 |
| Left medial orbitofrontal to right rostral middle frontal | 2.82 |
| Right inferior temporal to right transverse temporal | 2.82 |
| Right bankssts to right middle temporal | 2.81 |
| Left inferior parietal to right pars opercularis | 2.81 |
| Right caudal middle frontal to right pars opercularis | 2.81 |
| Left lateral orbitofrontal to right medial orbitofrontal | 2.80 |
| Left superior frontal to right caudal middle frontal | 2.76 |
| Left thalamus proper to right fusiform | 2.72 |
| Right pars triangularis to right precentral | 2.71 |
| Right lateral orbitofrontal to left frontal pole | 2.68 |
| Left superior frontal to right superior frontal | 2.68 |
| Left inferior parietal to right lingual | 2.66 |
| Left bankssts to left posterior cingulate | 2.65 |
| Right precentral to right superior frontal | 2.65 |
| Left amygdala to right posterior cingulate | 2.64 |
| Right lateral orbitofrontal to right rostral middle frontal | 2.63 |
| Right caudal anterior cingulate to right rostral anterior cingulate | 2.62 |
| Left inferior temporal to right hippocampus | 2.61 |
| Left lateral occipital to left amygdala | 2.60 |
| Right entorhinal to right middle temporal | 2.60 |
| Right pars orbitalis to right postcentral | 2.59 |
